# Development of New Hybrid Casein-Loaded PHEMA-PEGDA Hydrogels with Enhanced Mineralisation Potential

**DOI:** 10.3390/ma15030840

**Published:** 2022-01-22

**Authors:** Georgiana-Dana Dumitrescu, Andrada Serafim, Raluca-Elena Ginghina, Horia Iovu, Rodica Marinescu, Elena Olăreț, Izabela-Cristina Stancu

**Affiliations:** 1 Advanced Polymer Materials Group, Faculty of Applied Chemistry and Materials Science, University Politehnica of Bucharest, 011061 Bucharest, Romania; dumitrescugeorgiana93@yahoo.com (G.-D.D.); andrada.serafim0810@upb.ro (A.S.); horia.iovu@upb.ro (H.I.); elena.olaret@upb.ro (E.O.); 2 Research and Innovation Center for CBRN Defense and Ecology, 041327 Bucharest, Romania; ginghinaraluca@gmail.com; 3 Academy of Romanian Scientists, 050094 Bucharest, Romania; 4 Department of Orthopedics, University of Medicine and Pharmacy “Carol Davila”, 020021 Bucharest, Romania; rodicamarinescu@ymail.com

**Keywords:** hybrid, hydrogel, casein, mineralisation

## Abstract

Casein is a micellar protein rich in glutamic and aspartic acids as well as in phosphoserine. Considering its native affinity for calcium and the connection of sub-micelles through calcium phosphate nanoclusters, this protein holds promise for stimulating biomimetic mineralisation phenomena and direct binding with the mineral phase of hard tissues. In this work we prepared new hybrids based on casein embedded in a poly(2-hydroxyethyl methacrylate)-polyethyleneglycol diacrylate (PHEMA-PEGDA) hydrogel. The resulting materials were investigated structurally by Fourier transform infrared (FT-IR). Casein modified the water affinity and the rheological properties of the hybrids. The microstructure was explored by scanning electron microscopy (SEM) and the distribution of the protein was established by combined SEM micrographs and elemental mapping considering the casein-specific elements (P, N and S) not contained by the synthetic hydrogel matrix. The effect of casein on the mineralisation potential and stability of the mineral phase was investigated by FT-IR and SEM when alternating incubation in Ca/P solutions is performed. Increasing casein content in the hybrids leads to improved mineralisation, with localised formation of nanoapatite phase on the protein areas in the richest sample in protein. This behaviour was proved microstructurally by SEM and through overlapping elemental distribution of Ca and P from the newly formed mineral and P, S and N from the protein. This study indicates that nanoapatite-casein-PHEMA-PEGDA nanocomposites may be developed for potential use in bone repair and regeneration.

## 1. Introduction

The development of hybrid materials incorporating casein is gaining increasing attention considering the high availability and low cost of this protein and its interesting properties. The combination of synthetic hydrogels such as polyacrylamide (PAAm), poly(2-hydroxyethyl methacrylate) (PHEMA) and poly(sodium methacrylate) (PMANa) with casein has been performed to obtain hydrogels with added value when compared to individual ones [1,2,3,4,5,6]. This micellar protein is rich in glutamic and aspartic acids as well as in phosphoserine, these aminoacids presenting high affinity for calcium [3,4,5,6]. Casein micelles are naturally responsible for the prevention of calcification of mammary gland ducts, and they act as calcium delivery nanovehicles for physiological processes especially in neonates [6]. Moreover, the sub-micelles are connected through calcium phosphate nanoclusters [3,7]. Such a native proteic microstructure holds promise for stimulating biomimetic mineralisation and direct binding with the mineral phase of hard tissues. This hypothesis was early explored by our group by immobilizing casein into PHEMA hydrogels and it was found that only nanoapatite nucleation was stimulated after incubation in synthetic body fluid [3]. PHEMA is a biocompatible hydrogel widely used in tissue repair or reconstruction. It was previously reported that it does not promote efficient mineralisation although it is interesting for orthopaedic applications [1,8,9]. Different strategies to stimulate its capacity of forming mineral phase were therefore explored, including the immobilisation of alkaline phosphataze, carboxymethylation and the surface decoration with carboxyl-ended gold nanoparticles [1,8,9,10]. Moreover, since this hydrogel does not promote apatite formation, it was considered an ideal mineralisation-inert matrix to test the ability of casein to form or bind mineral structures when exposed to calcium and phosphate ions containing fluids, under physiological temperature and pH [3]. Synthetic hydrogels based on HEMA and poly(ethylene glycol) diacrylate (PEGDA) are also appealing as scaffolds for tissue reconstruction, being biocompatible and hydrophilic [11,12,13]. The present work investigates the synthesis of new casein-loaded hybrids in a PHEMA-PEGDA copolymer, with the aim of exploring the ability of the phosphoprotein to enhance mineral deposition in this synthetic hydrogel, after alternate incubation in Ca/P solutions.

## 2. Materials and Methods

### 2.1. Materials

2-hydroxyethyl methacrylate (HEMA) (Sigma Aldrich) was used as synthetic monomer and poly(ethylene glycol) diacrylate (PEGDA) with molecular weight of 575 (Sigma Aldrich, St. Louis, MO, USA) was used as a co-macromer and crosslinker. N, N-dimethyl-p-toluidine (N,N-DMpT) (Sigma Aldrich, St. Louis, MO, USA) and benzoyl peroxide (BPO) (Merck-Darmstadt, Germany) were used to initiate the polymerisation at room temperature. Casein (Sigma Aldrich, St. Louis, MO, USA) from bovine milk (technical grade) was used as protein phase and distilled water was prepared with a GFL apparatus. Calcium nitrate tetrahydrate (Ca(NO_3_)_2_·4H_2_O, 0.15M, pH = 9.0) (Merck), ammonium phosphate dibasic (NH_4_)_2_HPO_4_, 0.09M) (Merck, Darmstadt, Germania) and ammonium hydroxide solution NH_4_OH (Fluka, Seelze, Germany) were used to prepare calcium and phosphate baths needed for the alternate mineralisation treatment. Silver nitrate (Sigma Aldrich, St. Louis, MO, USA) was used to characterise the mineralisation of the studied hybrids by Von Kossa staining. Phosphate-buffered saline (PBS) pH 7.4 was supplied from Sigma Aldrich (St. Louis, MO, USA).

### 2.2. Preparation of Casein-Loaded Hybrid Hydrogels

The preparation of casein-loaded PHEMA-PEGDA hydrogels was performed by redox-initiated free radical bulk polymerisation at room temperature. The casein content was varied as described in Table 1, while the ratio between HEMA and PEGDA was maintained at 1:1 (*v*:*v*). The monomers were combined to form a liquid phase in which N,N-DMpT was added (0.1% molar to C=C double bonds from HEMA and PEGDA). Casein and BPO (1% molar to C=C double bonds from HEMA and PEGDA) were mixed and ground in a mortar until a fine powder was obtained. Then the powder was transferred into a polyethylene mould (with the base area 3 × 3 cm × cm) followed by addition of 1 mL of the liquid phase and spatula homogenisation (1 min). The polymerisation reaction was fast, the reaction is allowed to occur at 37 °C in an oven, for 4 h. The control hydrogel without casein was prepared, with the initiator dissolved in the monomer mixture. After the polymerisation, the hybrid hydrogels were removed from the moulds and immersed in distilled water to extract unreacted reagents (for a week). After this step, for further investigations, cylinders with diameters of 5 mm were cut with a sharp blade device. For rheological measurements, cylinders with diameters of 20 mm were cut. The samples were dried at 37 °C, in an oven.

### 2.3. Characterisation of Hybrid Hydrogels

#### 2.3.1. Gel Fraction Analysis

The gravimetric method was applied to measure the hydrogel’s gel fraction. The samples, as obtained from the polymerisation reaction, without purification, were placed in an oven at 37 °C to completely dry to constant mass. The dried samples were accurately weighed (W_i_) and then placed into 250 mL of deionised water to extract the water-soluble unreacted fraction. After a week, with water changed every 24 h, the specimens were removed from water and dried to constant weight (W_f_) at 37 °C. The gel fraction was calculated using Equation (1):

(1) $$\mathrm{Gel}\;\mathrm{fraction}=\frac{W_{f}}{W_{i}}\times 100$$

#### 2.3.2. Water Uptake Behaviour and Swelling in Phosphate Buffer Saline

Swelling studies were performed on hydrogels using dried specimens (approximately 2 mm height, diameter 5 mm), to determine if the presence of casein changes the liquid uptake when compared to the synthetic hydrogel without protein (H1P1C0). Prior to swelling, hydrogel dehydration was performed in an oven at 37 °C, for a week, and their mass was determined (W_dry_). Then, the samples were immersed in 10 mL distilled water (DW) and in 10 mL PBS pH 7.4, respectively, at 37 °C. The materials were removed from the testing fluid at predefined time intervals, t, and immediately wiped with filter paper to remove residual liquid and the wet mass was measured (W_wet_); then the samples were reimmersed in water and PBS. The procedure was repeated until the swelling equilibrium was reached. The swelling ratio (SR) was determined for each time point according to the well-known Equation (2) [14,15,16,17]:

(2) $$\mathrm{SR}\;\left(\%\right)=\frac{\left(W_{\mathrm{wet}}-W_{\mathrm{dry}}\right)}{W_{\mathrm{dry}}}\times 100$$

Maximum swelling degree (MSD) was considered the swelling ratio at equilibrium. The equilibrium liquid content (ELC) was calculated according to Equation (3), using W_wet_ values when equilibrium was reached:

(3) $$\mathrm{ELC}\;\left(\%\right)=\frac{\left(W_{\mathrm{wet}}-W_{\mathrm{dry}}\right)}{W_{\mathrm{wet}}}\times 100$$

The experiment was run in triplicate (n = 3) and the results were expressed as mean ± standard deviation (SD). Statistical analyses were performed using GraphPad Prism Software 6.0 (GraphPad Software Inc., San Diego, CA, USA). Statistical relevance was assessed with one-way ANOVA method, and Bonferroni post-test; statistical differences were considered significant for *p* < 0.05.

#### 2.3.3. Rheological Measurements

The influence of the casein loading ratio on the overall mechanical properties of the composites was evaluated through rheology tests using a Kinexus Pro rheometer (Malvern, Worcestershire, UK) equipped with a plate-plate geometry (upper plate diameter 20 mm). The oscillatory measurements were performed on a temperature interval ranging from 30–80 °C and a water lock was used to prevent dehydration during testing. Discs with the diameter of 20 mm were placed on the bottom plate of the equipment and the upper plate was lowered until a force of 0.5 N was achieved. To investigate the resistance to deformation of the synthesised composites, the complex modulus (G*, Pa) was monitored. The synthetic H1P1C0 sample (without casein in its composition) was used as control hydrogel.

#### 2.3.4. Mineralisation through Ca/P Alternate Incubation

The mineralisation treatment was applied using a modified version of the protocol reported in [18]. Briefly, the hybrids were first immersed in 0.09 M ammonium phosphate dibasic (NH_4_)_2_HPO_4_ aqueous solution for 30 min, followed by rinsing with distilled water. Then the samples were immersed in 0.15 M calcium nitrate tetrahydrate Ca(NO_3_)_2_‧4H_2_O aqueous solution, with pH adjusted to 9 with ammonium hydroxide (NH_4_OH) aqueous solution and maintained for another 30 min and again rinsed with distilled water. This two-step procedure represents a deposition cycle. Mineralisation treatment was applied to the hydrogels for one (samples denoted_1x) and respectively for three cycles (samples denoted_3x), as reported in [19]. The macroscopic investigation of the appearance of samples after the mineralisation treatment was first performed. Von Kossa staining is a qualitative method to visually detect calcium salts formation when the samples are treated with silver nitrate [20,21]. The samples washed and dried after incubation in Ca/P solutions, were immersed in 3 mL silver nitrate solution (1% by *w*/*v*) for 20 min and exposed to strong light, as described in [20,21]. Then they were extensively rinsed with distilled water and dried. Digital images were taken. Morphological and microstructural investigation was performed using scanning electron microscopy (SEM) as described below. Fourier transform infrared (FT-IR) spectra were also recorded using the equipment described below.

#### 2.3.5. Fourier Transform Infrared (FT-IR) Analysis

Fourier transform infrared (FT-IR) spectra were recorded using a JASCO 4200 spectrometer using with a Specac Golden Gate attenuated total reflectance (ATR) device in the 4000–600 cm^−1^ wavenumber region, with a resolution of 4 cm^−1^ and an accumulation of 200 spectra. Casein, H1P1C0 synthetic hydrogel, and nanohydroxyapatite (from Sigma) were used as control samples.

#### 2.3.6. Morpho-Structural Characterisation

Morphological and microstructural characterisation of the hydrogels was performed through scanning electron microscopy (SEM) using a Tescan Vega II LMU SEM at an acceleration voltage of 30 keV, at high vacuum: 3.4 e-002 Pa, using a SE detector and VegaTC software. The device was equipped for EDS microanalysis with a Bruker Quantax XFlash 6/10 energy-dispersive X-ray spectrometer. Top view and side view imaging, elemental mapping, spectrum quantification with P/B-ZAF were performed using Esprit software. The cylindrical samples (5 mm diameter) were prepared for topography and cut with a sharp blade for cross section specimen (approximately 2 mm thick) and then mounted on the metal stub with carbon disc to enhance conductivity. For each composition, the samples were coated on Quorum minisputter SC7620 with Au/Pd under Ar plasma, for 3 min, process current: 1.5 mA, chamber pressure: 2 × 10^−1^ mbar prior to analysis. Micrographs were recorded for topography and, respectively, cross section examination.

## 3. Results and Discussions

### 3.1. Preparation and Characterisation of Hybrid HPC Materials

**Figure 1:**
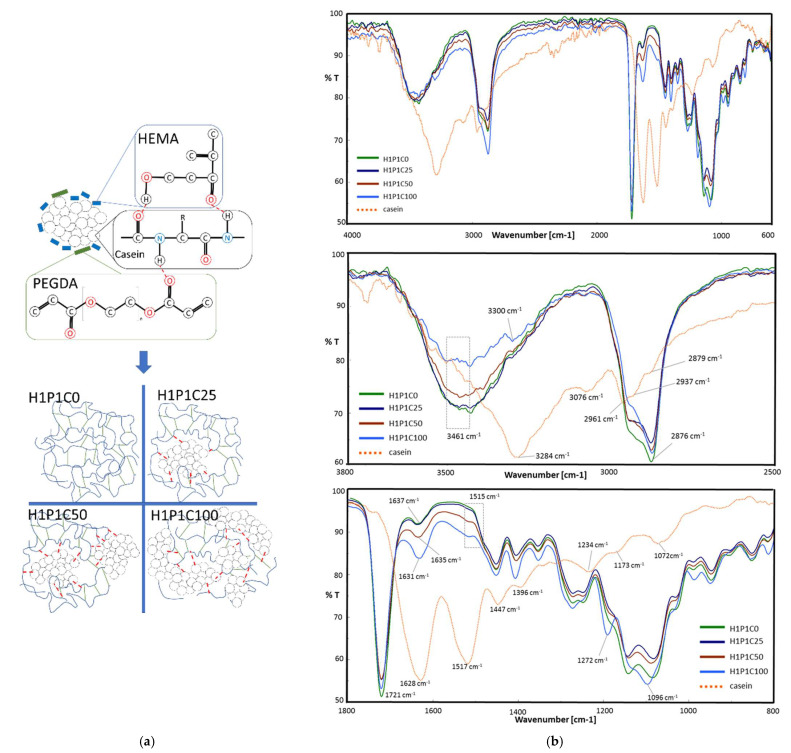
Hybrids synthesis. (**a**) Schematic representation of the hybrids synthesis: Casein (C) micelles dispersed in HEMA (H)-PEGDA (P) polymerisation mixture, with a molar ratio of 4:1. Hydrogen-bond formation between Casein and co-monomers (red dotted line); polymerisation products: synthetic copolymer H1P1C0 and casein-loaded hydrogels with increasing protein content H1P1C25, H1P1C50, H1P1C100; (**b**) from top to down: whole range FT-IR spectra of the hybrids with different casein content; FT-IR spectral details of 2 representative domains: 3800–2500 cm^−1^ and 1800–800 cm^−1^. Casein and H1P1C0 are used as control samples.

This study aimed, in the first step, to obtain new hybrid hydrogels based on casein embedded into a synthetic HP matrix, further denoted H1P1C hydrogels. We previously explored casein-PHEMA hybrids for their mineralisation potential in synthetic body fluid, the hybrid hydrogels being obtained by network-forming polymerisation in the presence of commercial PHEMA macromolecules and protein dissolved in NaOH solution, through crosslinking of HEMA with 3% molar tetraethylene glycol dimethacrylate [1]. Although the macromolecular matrix obtained was not dense, due to already existing PHEMA polymer only mineral nucleation was noticed at immersion in synthetic body fluid. Therefore, we decided to investigate in the present work a new PHEMA copolymer hydrogel, with a higher crosslinking degree due to 20% crosslinker to HEMA monomer. Moreover, the reaction mixture is not supplemented with PHEMA macromolecules and casein was not dissolved anymore, and the polymerisation occurred through free radical bulk polymerisation. The densely crosslinked copolymer was combined directly with casein aggregates and submitted to alternating incubation in calcium and phosphate solutions to induce apatite-like precipitation, with the aim of investigation if casein has the ability to stimulate mineral deposition and binding in PHEMA copolymers. In the present paper, the macromolecular matrix of the hybrids is represented by a synthetic hydrogel prepared through the network-forming radical copolymerisation of HEMA and PEGDA. The comonomers were combined in this research in a HEMA:PEGDA 4:1 molar ratio, as suggested in Figure 1a, being distributed around casein aggregates with increasing concentration. The control hydrogel H1P1C0 was obtained similarly but without the protein aggregates. This corresponded to a dense network, containing 20% molar PEGDA crosslinker to HEMA monomer. When PHEMA hydrogels were typically prepared using short chain ethylene glycol dimethacrylate crosslinkers such as ethylene glycol dimethacrylate and tetraethylene glycol dimethacrylate, a ratio below 3% molar was typically used, leading to hydrogels with a lower crosslinking density [1,8,9,10]. For the hybrids, the preparation strategy involved the network-forming polymerisation of the synthetic comonomers in the presence of casein aggregates. First, the polymerisation system was homogenised and hydrogen-bonds between the protein micelles and the synthetic molecules were expected to occur, as suggested in Figure 1a. Briefly, a combination of OH···O=C, OH···NH and C=O···HN between OH and C=O of HEMA and PEGDA molecules and amide bonds and side groups of casein was expected to occur, in a further reactive microenvironment leading to formation of hydrogel network embedding the protein micelles. Similar physical interactions were described for hybrid structures obtained by network forming polymerisation of sodium-neutralised methacrylic acid in the presence of sodium caseinate [5].

The structure of the hybrids was assessed by FT-IR and representative spectra are presented in Figure 1b. The spectrum of casein presents typical vibrations for proteins, with a strong and broad amide A peak at 3284 cm^−1^, an amide B peak at 3076 cm^−1^ and a doublet at 2961 and 2937 cm^−1^ corresponding to saturated C-H stretching. The two major bands of the protein backbone are amide I at 1628 cm^−1^ and amide II at 1517 cm^−1^, while amide III is visible at 1234 cm^−1^. At 1447 cm^−1^ the bending vibration of methylene group was noticed. The stretching vibration at 1072 cm^−1^ is assigned to symmetric stretching of HPO_4_^2−^ anion, and the band at 922 cm^−1^ is attributed to P–OH vibration. Similar spectra analysis was reported in [2,5,22,23,24]. The control synthetic H1P1C0 copolymer presented O-H stretching vibration at 3462 cm^−1^, C-H doublet at 2937 and 2876 cm^−1^ and a strong ester carbonyl vibration at 1721 cm^−1^ from the two types of structural units, HEMA and PEGDA [16].

The presence of casein in the hybrid hydrogels induced some spectral modification. First, it broadened, reduced the peak height and slightly downshifted the O-H stretching vibration. A new peak (visible as a shoulder) at about 3300 cm^−1^ appeared due to the amide A from the protein. This peak increased with the casein content, and it is upshifted when compared to amide A in protein, suggesting hydrogen-bonding with O-H from HEMA structural units. New weak amide B vibrations were visible in the spectra of the hybrids at about 3080 cm^−1^. Furthermore, the major C=O vibration of the synthetic polymer matrix remains at 1721 cm^−1^ while its intensity is slightly decreasing with increasing the protein content. Increasing casein content led to stronger amide I and amide II vibrations, little upshifted with respect to the corresponding bands in pristine protein. Accordingly, in H1P1C100, amide I appeared at 1631 cm^−1^ (little upshift with respect to peak position in casein) and amide II at 1515 cm^−1^ (scarcely downshifted against casein). Such spectral changes may be assigned to protein C=O and N-H involvement in hydrogen-bonding with the O-H and C=O of HEMA and PEGDA structural units of the synthetic matrix, as schematically suggested in Figure 1a. Hydrogen-bonding between casein groups and O-H or -C=O bearing polymers have been previously reported [5]. In our work, it must be stated that the micellar structure of the protein was not destroyed by NaOH treatment and therefore, hydrogen-bonding was expected to occur only at the interface between the synthetic molecules and the protein micelles surface layer. These interactions stimulated the polymerisation to occur between HEMA monomer and PEGDA macromer as they were placed around the micellar structures of the protein. This was why the intensity of hydrogen- interactions in the final hybrids was more reduced that, for example, in hybrids based on neutralised poly(methacrylic acid) and sodium caseinate leading to higher vibrations shifting [5]. Casein-based PHEMA-PEGDA hybrids with increasing casein content present a band with increasing intensity at 1096 cm^−1^, with the highest contribution of phosphate vibration in H1P1C100. The importance of phosphate groups was acknowledged for the organisation of protein in micelles. The presence of phosphate-specific vibrations in the hybrids indicates that casein keeps its micellar structure during the polymerisation reaction. Other works reported the disappearance of phosphate vibrations corresponding to the loss of micellar structure of sodium caseinate, when immobilised in sodium poly(methacrylate) matrix [5].

**Figure 2:**
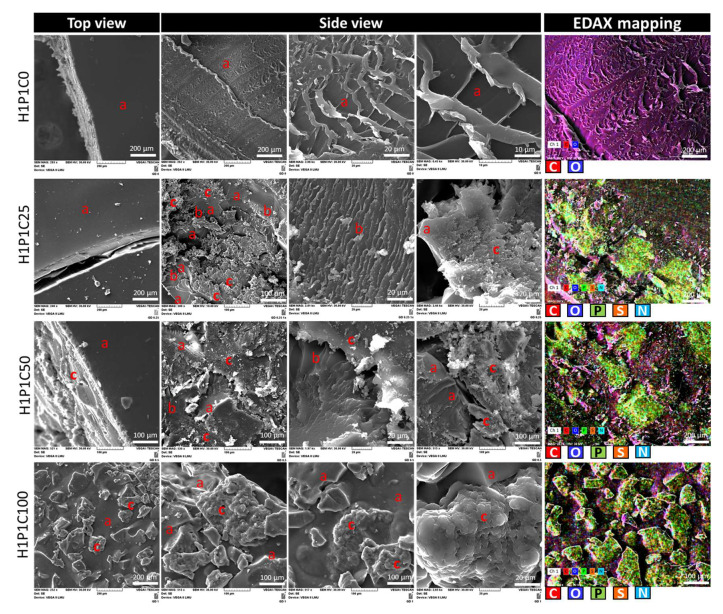
Representative SEM micrographs revealing microstructural features of the H1P1C25/50/100 hybrids with increasing casein content. Amorphous H1P1C0 hydrogel was used as a control. First image in each raw—top view; the other three SEM micrographs display side view images taken from the cross-sections (a—amorphous synthetic hydrogel, b—porous interface between synthetic and casein, c—casein micelles); 5th column—representative EDAX mapping showing the distribution of casein (overlapping areas rich in N, P and S) in the synthetic matrix containing C and O (cross-section view, colour code assigned for each representative element).

To better understand the new casein-based hydrogels, their microstructural features were explored by SEM as presented in Figure 2. The synthetic hydrogel H1P1C0 presented a smooth surface (H1P1C0 top view in Figure 2) and a rather compact amorphous phase in bulk (H1P1C0 top view in Figure 2). Such morphology was specific to PHEMA hydrogels as also reported in [10,25]. The hybrid scaffolds presented an amorphous synthetic polymer matrix (denoted with a in Figure 2) embedding islands of protein micelles (denoted with c in Figure 2) with a specific granular protein microstructure. The interface area between these two phases presents an interesting appearance, with a lamellar-like organisation (b in Figure 2), that may result from a progressively decreasing water-affinity from the casein micelles to the synthetic polymer, considering that the samples were extracted in water after their synthesis and the solvent was removed by in-air dehydration, at 37 °C. Increasing casein content led to a modification of the scaffold morphology. When analysing the top view appearance using SEM, the hydrogels H1P1C25 and H1P1C50 presented a relatively smooth amorphous surface (indicated with (a) in the corresponding top view images from Figure 2) similar to that of the H1P1C0 control hydrogel. Only isolated casein micelles were noticed. The sample with the highest casein content, H1P1C100, presented a biphasic rough topography, with numerous protein micelles exposed to the surface (c area in H1P1C100 top view section of Figure 2), and a similar biphasic bulk microstructure. The granular morphology of casein aggregates (areas c in Figure 2) was similar with that reported in other works such as for commercial casein in [1,7]. Each micellar cluster was rough due to agglomerations of sub-micelles (areas c in Figure 2). A detail of a casein cluster identified in H1P1C100 is given in Figure 2 (3rd micrograph from left to right in H1P1C100 side view). It was reported in [7] that a cluster of calcium phosphate may be involved in the aggregation of sub-micelles. Furthermore, SEM micrographs revealed specific biphasic appearance of the hybrids cross-sections. H1P1C25 presented protein micellar domains dispersed in the amorphous hydrogel, with an interesting porous synthetic-protein interface layer (area 2) that often forms parallel or slightly divergent lamella/walls. The distribution of casein in the synthesised hybrids was also confirmed by element mapping as shown in Figure 2, taking advantage of the protein diagnose elements: P from phosphoryl serine, N from the amide and amine groups and S from the two cysteines that form intermolecular disulphide bridges [7]. To the best of authorsʹ knowledge, such characterisation has not been previously reported for casein or casein-loaded synthetic hybrids. The synthetic polymer matrix presented C and O, while commercial casein contains, in addition to C and O, three specific elements P, S (from cysteine) and N. EDAX mapping of the control hydrogel exclusively identified C and O as shown in Figure 2. For the hybrid samples, an overlapping of individual elemental mappings was noticed that allowed us to identify areas with colocalised P, N and S, surrounded by a matrix rich in C and O. Such distribution was assigned to casein aggregates phase dispersed in the synthetic hydrogel. Figure 2 also proves that for the investigated hybrids, the phase distribution according to the elemental maps corresponded to that obtained through morphology evaluation in secondary electrons mode.

**Figure 3:**
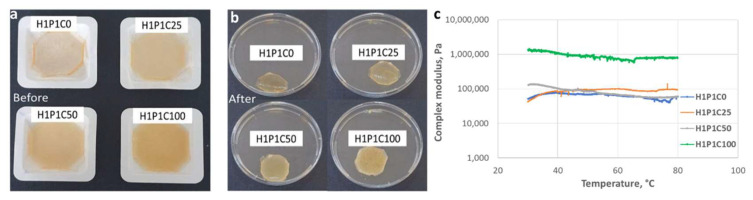
Samples (**a**) before and (**b**) after purification. (**c**) Rheological behaviour of H1P1C25/50/100 hybrids versus H1P1C0.

The hydrogels macroscopic appearance is presented in Figure 3a,b. The control synthetic hydrogel was glassy and transparent while enhancing protein content in the studied hybrids reduced the transparency and increased the elasticity of the samples. It was previously reported that casein is in its rubbery state at room temperature, having a glass transition temperature below room temperature [7]. No significant visible material loss or fragmentation were noticed after extraction in water. The gel fraction was calculated using Equation (1). GF values above 90% were obtained, stating for an efficient polymerisation and casein immobilisation.

The rheological data showed that a casein loading below 50% with respect to the synthetic matrix has little influence on the overall rigidity of the material, regardless of the temperature value. Figure 3c, showing the influence of casein addition on the modification of the complex modulus in the 30–80 °C interval was representative with this respect considering that the complex modulus indicated the resistance to deformation of the investigated material. However, slight differences in the composites’ behaviour can be noticed in the temperature range 30–40 °C. The control sample H1P1C0 and the sample with the lowest protein loading ratio, (H1P1C25) exhibited a stiffness, while a loading ratio of 50 and 100% led to materials with increased elasticity. For samples H1P1C50 and H1P1C100, this behaviour was maintained throughout the entire studied temperature range, while for samples H1P1C25 and H1P1C0 the increase of elasticity with increasing temperature was showed only above 40 °C.

**Figure 4:**
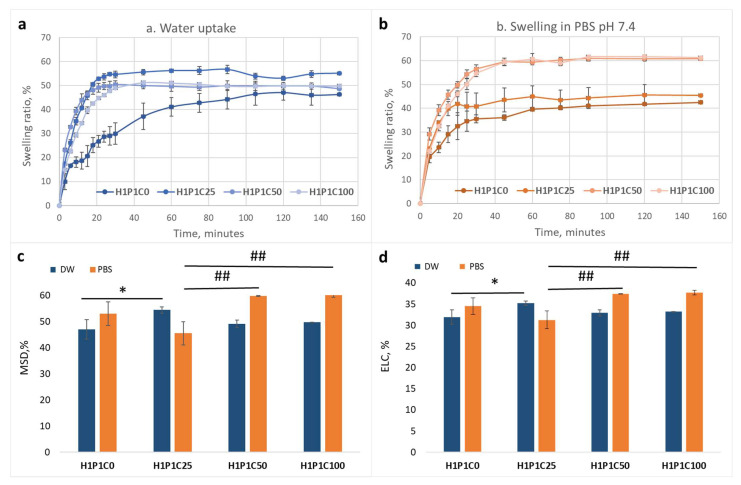
Influence of casein content on: (**a**) water uptake; (**b**) swelling behaviour in PBS pH 7.4; (**c**) maximum swelling degree, MSD, in DW and PBS pH 7.4; (**d**) equilibrium liquid content, ELC, in DW and PBS pH 7.4 (statistical significance: * *p* < 0.05, for DW; statistical significance: ## *p* < 0.01, for PBS).

Figure 4 presents the effect of casein loading on the water and PBS affinity of the hybrids when compared to the synthetic hydrogel. It is visible in Figure 4a that the addition of casein in all hybrids increased the hydration rate, with the maximum swelling degree (determined as the maximum swelling ratio) being reached faster, in less than 20 min), than for the control H1P1C0 requiring approximately 100 min. This was most probably due to the porous and hydrophilic casein micelles that enhanced faster water uptake. A similar behaviour was noticed also at incubation in PBS pH 7.4, as visible in Figure 4b. It may be noticed that the control H1P1C0 hydrogel reached maximum swelling faster than in distilled water, namely in about 80 min, and slower than the casein-based hybrids reaching equilibrium in PBS in about 40 min. While H1P1C0 swelled faster in PBS than in water, the casein hybrids got equilibrium swelling in PBS slower than in water.

Interestingly, adding 25 wt% casein in the hybrid lead to a slightly higher maximum swelling degree when compared to both control and the two other hybrids richer in casein (Figure 4c). The equilibrium water content was also higher for sample C25 (Figure 4d). Increasing protein content in the hybrids, led to slightly higher affinity for both water and PBS (Figure 4). This may also be responsible for the elasticity increase noticed rheologically.

### 3.2. Casein Effect on the Mineralisation of the Hybrids

Our team was previously interested in exploring the potential of anionic species including casein to stimulate the mineralisation of synthetic polymers resulting in hybrids with application in bone or dental reconstruction or regeneration [1]. It was shown, under acellular in vitro conditions, at physiological pH and temperature, that this protein has the capacity to form apatite nucleation areas when immobilised in PHEMA and incubated in synthetic body fluid for two weeks [1]. In that research, sodium caseinate (casein dissolved in NaOH solution) was embedded in a matrix represented by PHEMA hydrogel obtained through polymerisation of HEMA with a shorter crosslinking agent, tetraethylene glycol dimethacrylate, in the presence of PHEMA macromolecules [1]. Only 3% (molar) crosslinker was used when compared to 20% PEGDA in the present work, making the network of H1P1C hydrogels denser. Furthermore, the micellar microstructure of this calcium-binding protein was reported to result from aggregation of sub-micelles connected to clusters of calcium phosphate [7]. The present study aimed at exploring if casein would improve mineral coating and loading when the protein was immobilised into synthetic hydrogels not presenting mineralisation potential. Therefore, to produce the mineral phase, alternating incubation in calcium- and phosphate-solutions was performed, at 1 or 3 cycles, as similarly reported [19]. The samples were investigated first for macroscopic changes and then by Von Kossa staining, SEM with EDAX and FT-IR.

**Figure 5:**
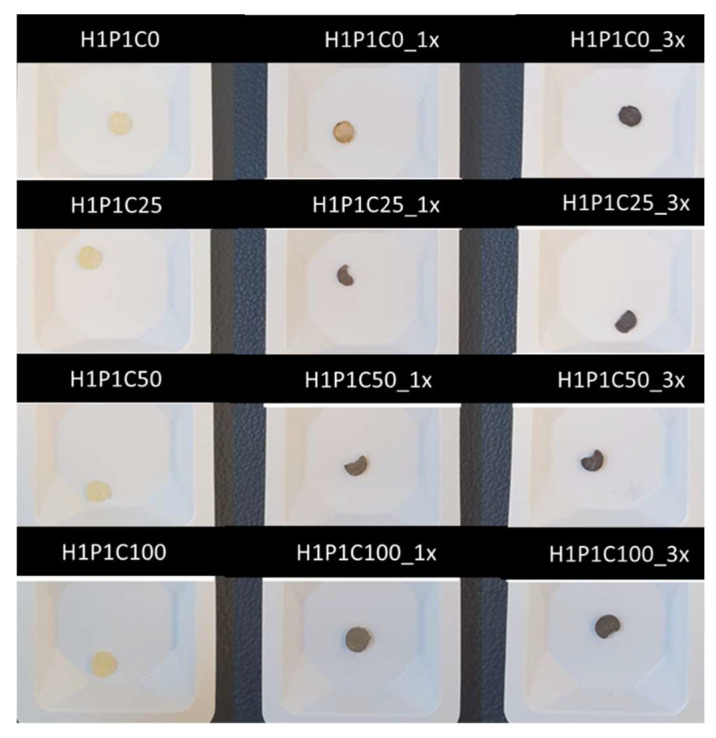
Representative digital images of samples after Von Kossa staining applied to unmineralized hydrogels (H1P1C) and to hydrogels treated with one Ca/P cycle (H1P1C_1x), and three cycles (H1P1C_3x).

The hybrids appearance changed following soaking in Ca/P, with higher opacity corresponding to samples richer in casein and incubated for three cycles. Von Kossa staining revealed different colour intensity depending on the casein content (Figure 5). The samples not submitted to mineralisation cycles kept their original appearance, with no brown colour detected. For the hydrogels incubated for 1 cycle in Ca/P baths, the brownish colour intensity stating for identified calcium salts increased with the protein loading content. This suggested that calcium salts formation during soaking in Ca/P is stronger for casein-rich materials. After one cycle of incubation, the control hydrogel only presented a little colour change, when compared to its initial appearance, most probably due to calcium phosphate entrapped into the hydrogel network without formation of a mineral phase. The staining intensified, as expected, after three cycles of incubation suggested a potential stronger deposition of a Ca/P mineral phase. Similar results were reported in the literature, with PHEMA remaining translucent and carboxymethylated PHEMA becoming dark brown when biomimetically mineralised in synthetic body fluid [10].

**Figure 6:**
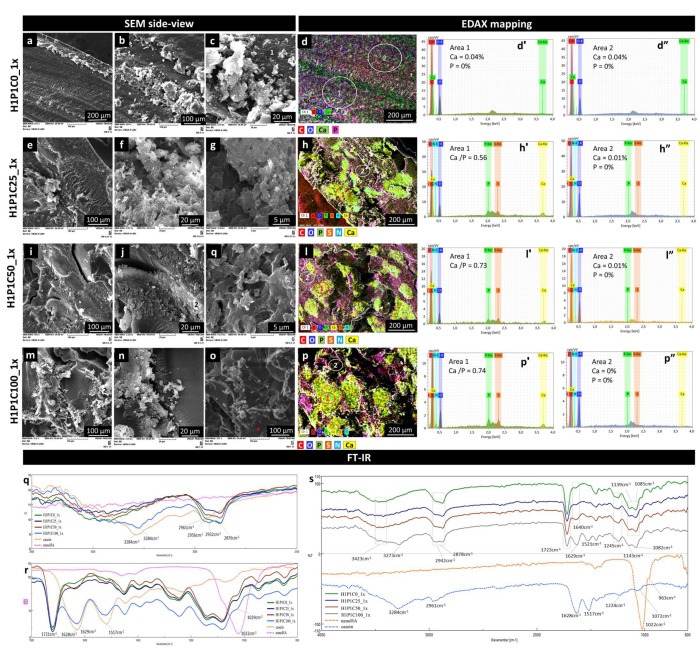
Representative SEM micrographs (**a**–**c**,**e**–**g**,**i**–**k**,**m**–**o**) and EDAX mapping (**d**,**h**,**l**,**p**) exploring microstructural changes after 1 cycle of mineralisation: H1P1C25, H1P1C50, H1P1C100 hybrids with increasing casein content. H1P1C0 hydrogel was used as a control; individual colour codes are displayed for elements identified by EDAX; representative EDAX spectra for mineralised area 1 in the microelemental maps (**d’**,**h’**,**l’**,**p’**) and for hydrogel matrix in area 2 in the microelemental maps (**d”**,**h”**,**l”**,**p”**); FTIR spectra recorded onto the surface: wavenumber interval 3000–2500 cm^−1^ (**q**), wavenumber interval 1800–800 cm^−1^ (**r**) and whole range spectra (**s**).

SEM analysis revealed that the general morphology of the hybrids was mainly kept after 1x incubation. Some cracks were due to drying. Figure 6 presents representative side view micrographs proving the formation of Ca/P mineral phase for all the protein-containing samples. The control synthetic hydrogel presented minor morpho-structural changes (general view in Figure 6a, and details in Figure 6b,c). EDAX mapping in Figure 6d and the corresponding EDAX spectra indicated that the H1P1C0_1x hydrogel seems to contain only Ca in addition to C and O. Addition of the protein in the hybrids was associated with enhanced formation of Ca/P containing mineral phase. For H1P1C25_1x, a biphasic appearance was noticed, with only few and small mineral structures localised on the casein micelles (Figure 6e–g). The mineral structures were a bit more numerous in H1P1C50_1x and H1P1C100_1x, as visible in Figure 6, also localised in areas containing casein micelles. The mineral seemed freely growing, probably in an incipient form of development, without the specific organisation in semispheroidal grains typically reported in strong mineralizing materials based on PHEMA materials activated for mineralisation [8,10,25,26]. EDAX analysis indicated that the distribution of Ca and P overlapped with the distribution of the elements specific to protein (S, N and P) (Figure 6h,l,p). Corresponding EDAX spectra identified in area 1, Ca/P ratio of 0.56 for H1P1C25_1x and Ca/P ratio of 0.73 and 0.74 for H1P1C50_1x and H1P1C100_1x. These data indicated increasing calcium binding with increasing casein content. Negligible P and Ca were identified on the hydrogel matrix (area 2). Altogether, these findings bring strong evidence supporting the initial hypothesis that the protein had the capacity of stabilizing the mineral formation in synthetic hydrogels, while the hydrogel itself was not sustaining mineral deposition.

Additional details were investigated by FTIR spectra (Figure 6q–s). In Figure 6q, the spectra of the hybrids present a major broad vibration specific to O-H from the synthetic polymer combined with amide A from casein. Following the soaking cycle in Ca/P solutions, few spectral changes were noticed when compared to spectra of as-synthesised hybrids in Figure 1. The peak broadened and the maximum was upshifted. The strongest modification is visible for H1P1C100_1x, with a shoulder at about 3400 cm^−1^ assigned to O-H from the synthetic hydrogel and a maximum at 3284 cm^−1^ corresponding to amide A from casein, after 1x incubation. In Figure 6r, the C=O from synthetic hydrogel is visible at 1721 cm^−1^, amide I and II at 1629 cm^−1^ and 1517 cm^−1^ increased in intensity with increasing casein content. No significant new peak corresponding to a mineral phase containing phosphate was noticed around 1022 cm^−1^, probably due to an insufficient mineralisation at the surface of the hybrids.

**Figure 7:**
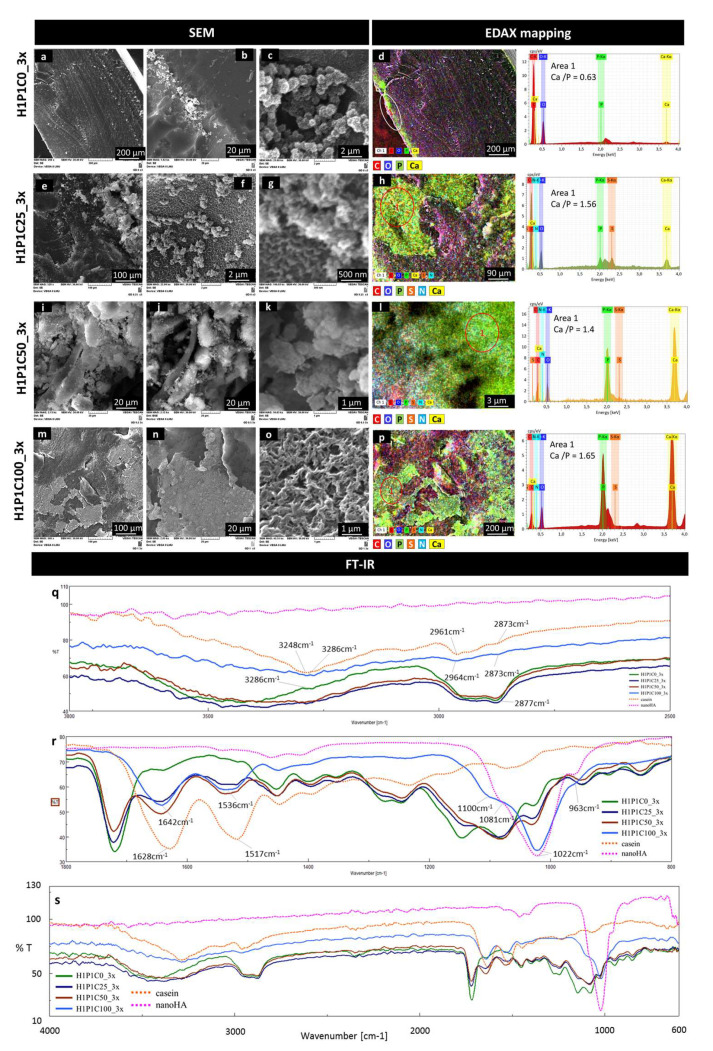
Representative SEM micrographs (**a**–**c**,**e**–**g**,**i**–**k**,**m**–**o**) and EDAX mapping (**d**,**h**,**l**,**p**) exploring microstructural changes after 3 cycles of mineralisation: H1P1C25, H1P1C50, H1P1C100 hybrids with increasing casein content, and representative EDAX spectra of the mineralised areas 1. H1P1C0 hydrogel was used as a control; representative FT-IR spectra recorded onto the surface: wavenumber interval 3000–2500 cm^−1^ (**q**), wavenumber interval 1800–800 cm^−1^ (**r**), whole range 4000–600 cm^−1^ (**s**).

The results of the incubation of the samples for three cycles, showed additional data on the mineralisation. Figure 7 is representative with this respect. The micrographs from Figure 7a,b show that on the control H1P1C0_3x hydrogel, only modest Ca/P areas were formed in the proximity of the surface, similar to the localisation identified after 1 cycle of incubation. Although the surface of the sample did not contain well-structured mineral deposits (Figure 7b), clusters of freely grown globular nanospheroids were formed (Figure 7c). They were monodispersed dimensionally, with diameters around 1 µm and with a microstructure consisting of radially disposed nanometric needles and plates assigned in other works to apatite-like nanostructured mineral crystals [8,10,19,25,26]. These mineral deposits formed chain-like structures and were not embedded in the synthetic hydrogel but rather emerging from the surface. The EDAX map in Figure 7d confirmed Ca/P presence as sub-surface layer, with a Ca:P ratio of 0.63. These results suggest that this new PHEMA-based copolymer was not supporting mineralisation and did not strongly bind to mineral deposits formed by alternating Ca/P incubation. Other PHEMA scaffolds obtained with shorter crosslinkers such as ethylene glycol dimethacrylate and tetraethylene glycol dimethacrylate only mineralised in synthetic body fluids when seeded or coated with anionic components such as through carboxymethylation, copolymerisation with 2-methacryloylamido glutamic acid, coating with succinamic acid-gold nanoparticles or immobilisation of alkaline phosphatase [8,10,25,26].

The morpho-structural changes in the hybrid casein-containing scaffolds were significant after the 3x Ca/P incubation. H1P1C25_3x presented mineral aggregates, with semi-spheroidal shape, with diameters below 1 µm, also formed by needle- or plate-like crystals with radial disposition. The semi-spheroidal shaped mineral deposits were well anchored onto the hybrid suggesting the mineral was formed from the hydrogel (Figure 7e,f), and not just precipitated through a local accumulation of ions. Figure 7g presents a micrograph with aggregated semispheroidal structures with morphology specific to nanostructured apatite-like mineral, developed from a nanostructured hydrogel matrix with morphology specific to casein micelles (scalebar 500 nm). The EDAX mapping indicated that these aggregates are Ca/P rich, with a Ca/P ratio of 1.56, while they were overlapped onto areas rich in P, S and N, assigned to casein micelles (Figure 7h). No Ca/P deposits were formed on the synthetic matrix corresponding to the C,O-rich area (Figure 7h). Sample H1P1C50-3x induced the formation of apatite-like crystalline structures. Figure 7i,j shows a mineral rich deposition with specific nanoapatite-like morphology, as revealed by the appearance in secondary electrons (Figure 7i) and backscattered electrons (Figure 7j). The crystalline needles seem densely packed and strongly adherent to the hybrid material (Figure 7k), suggesting a higher compatibility between the newly formed mineral phase and the scaffold. The EDAX map in Figure 7l indicates a perfect overlapping between individual maps specific to Ca, P, S and N, localizing again the mineral formation on the casein islands. The Ca/P ratio on the mineralised area was around 1.4. While H1P1C25_3x and H1P1C50_3x only presented mineral structures in the bulk of the hybrids and not exposed to the surface, the richest sample in protein, H1P1C100_3x, was covered by compact mineral areas (Figure 7m,n). The micrograph in Figure 7o presents the morphological details specific to nanoapatite crystals intimately combined with the organic matrix. We previously described similar morphology consisting in bundles of rod-like nanocrystallites generating a porous nanocomposite structure [27]. Such morphology suggested that the mineral was formed following intense and homogeneous Ca/P capturing by the hydrogel, followed by precipitation. The EDAX map from Figure 7p associates the newly formed mineral, corresponding to Ca and P-rich areas, with the protein aggregates assigned to overlapping S, N maps. While the EDAX spectra were recorded, representative Ca/P ratio of 1.65 were obtained on the mineralised area at the surface of the hybrids (EDAX spectrum for area 1 in Figure 7p). This suggests that exposed casein islands on H1P1C100_3x were coated with apatite.

FT-IR analysis provided spectral evidence on a stronger mineral formation after 3 cycles when compared to 1 cycle of Ca/P soaking. The intensity of the combined broad peak assigned to O-H and amide A and B is decreasing, as well as the C-H vibrations (Figure 7q). The strongest modification was noticed for the casein-richest hybrid, H1P1C100_3x. Furthermore, the C=O and amide I and amide II vibrations specific to the synthetic hydrogel and to casein also decrease in intensity. The most important spectral change for the hybrids was an increase in the intensity of the shoulder at about 1022 cm^−1^ (Figure 7r). Sample H1P1C100_3x presents a significantly modified spectrum, with a new strong trimodal peak, centred at 1022 cm^−1^ and with shoulders at 963 cm^−1^ and at 1100 cm^−1^, assigned to specific phosphate vibrations. This was similarly to the phosphate vibration of nanoapatite (nanoHA) used as a control (Figure r). Moreover, the spectrum of H1P1C100_3x did not contain the peak corresponding to C=O vibration from the synthetic component. This suggests the sample was coated by a strong layer of mineral spectrally detected considering the FTIR analysis was performed using the ATR mode. This was in agreement with the fact that casein micelles were exposed only to the surface of the H1P1C100 hybrids, as shown in Figure 2. Altogether, these results describe a more intense mineralisation with increasing casein content in the hybrid hydrogels. Moreover, the mineralisation occurrence was proved to be localised onto the casein micellar aggregates.

Furthermore, the mineralisation of poly(2-hydroxyethyl methacrylate) (PHEMA) was previously investigated and, since the homopolymer does not have the potential to efficiently mineralise for bone binding uses when incubated in synthetic plasma, hydroxyapatite nodules, denoted as calcospherites, were developed, that is PHEMA-alkaline phosphatase hybrids [9]. We previously reported the potential of casein to be used as nanosised anionic nucleator for biologically apatite induction when immobilised in poly(HEMA) [1]. The investigated hybrids only nucleated Ca-P mineral phase after two-weeks incubation of synthetic body fluid. We showed that the size and density of the formed nodules was dependent on the protein:PHEMA ratio. The synthetic hydrogel was obtained through the free radical network-forming copolymerisetion of HEMA with tetraethyleneglycol dimethacrylate in the presence of commercial PHEMA [1]. In the present work, we prepared a synthetic HEMA-based hydrogel using a longer molecule comonomer acting as a crosslinker, PEGDA. This was done to modify the hydrogel microenvironment in which the micellar protein was embedded. The morphology of the HEMA-PEGDA-casein hybrids was biphasic, with denser casein aggregates in the protein richer materials, similarly to that reported for PHEMA-casein [1].

## 4. Conclusions

A new family of biphasic casein-loaded hydrogels was prepared using PHEMA-PEGDA as a synthetic matrix. This work demonstrated that casein micellar aggregates enhance mineral deposition when alternating incubation in Ca/P solutions is performed. Increasing casein content leads to an intimate combination of bundles of rod-like nanoapatite crystals with the protein superficial layer, in H1P1C100. This opens the way in developing nanoapatite-casein-PHEMA-PEGDA nanocomposites with potential applications in bone repair and regeneration.

## Figures and Tables

**Table 1:** Casein content of polymerisation mixtures.

Sample	Casein (wt%)
H1P1C0	0
H1P1C25	25
H1P1C50	50
H1P1C100	100

## Data Availability

The data presented in this study are available on request from the corresponding author.
